# Animal-related factors associated with moderate-to-severe diarrhea in children younger than five years in western Kenya: A matched case-control study

**DOI:** 10.1371/journal.pntd.0005795

**Published:** 2017-08-04

**Authors:** Anne Conan, Ciara E. O’Reilly, Eric Ogola, J. Benjamin Ochieng, Anna J. Blackstock, Richard Omore, Linus Ochieng, Fenny Moke, Michele B. Parsons, Lihua Xiao, Dawn Roellig, Tamer H. Farag, James P. Nataro, Karen L. Kotloff, Myron M. Levine, Eric D. Mintz, Robert F. Breiman, Sarah Cleaveland, Darryn L. Knobel

**Affiliations:** 1 Ross University School of Veterinary Medicine, Basseterre, St Kitts and Nevis; 2 Division of Foodborne, Waterborne, and Environmental Diseases, US Centers for Disease Control and Prevention, Atlanta, Georgia, United States of America; 3 School of Health Sciences, Jaramogi Oginga Odinga University of Science and Technology, Bondo, Kenya; 4 Kenya Medical Research Institute, Centre for Global Health Research, Kisumu, Kenya; 5 Division of Global Health and Protection, Center for Global Health, US Centers for Disease Control and Prevention, Atlanta, Georgia, United States of America; 6 Center for Vaccine Development, University of Maryland School of Medicine, Baltimore, Maryland, United States of America; 7 International Emerging Infections Program, Centers for Disease Control and Prevention, Nairobi, Kenya; 8 Institute of Biodiversity, Animal Health and Comparative Medicine, College of Medical, Veterinary and Life Sciences, University of Glasgow, Glasgow, United Kingdom; Massachusetts General Hospital, UNITED STATES

## Abstract

**Background:**

Diarrheal disease remains among the leading causes of global mortality in children younger than 5 years. Exposure to domestic animals may be a risk factor for diarrheal disease. The objectives of this study were to identify animal-related exposures associated with cases of moderate-to-severe diarrhea (MSD) in children in rural western Kenya, and to identify the major zoonotic enteric pathogens present in domestic animals residing in the homesteads of case and control children.

**Methodology/Principal findings:**

We characterized animal-related exposures in a subset of case and control children (n = 73 pairs matched on age, sex and location) with reported animal presence at home enrolled in the Global Enteric Multicenter Study in western Kenya, and analysed these for an association with MSD. We identified potentially zoonotic enteric pathogens in pooled fecal specimens collected from domestic animals resident at children’s homesteads. Variables that were associated with decreased risk of MSD were washing hands after animal contact (matched odds ratio [MOR] = 0.2; 95% CI 0.08–0.7), and presence of adult sheep that were not confined in a pen overnight (MOR = 0.1; 0.02–0.5). Variables that were associated with increased risk of MSD were increasing number of sheep owned (MOR = 1.2; 1.0–1.5), frequent observation of fresh rodent excreta (feces/urine) outside the house (MOR = 7.5; 1.5–37.2), and participation of the child in providing water to chickens (MOR = 3.8; 1.2–12.2). Of 691 pooled specimens collected from 2,174 domestic animals, 159 pools (23%) tested positive for one or more potentially zoonotic enteric pathogens (*Campylobacter jejuni*, *C*. *coli*, non-typhoidal *Salmonella*, diarrheagenic *E*. *coli*, *Giardia*, *Cryptosporidium*, or rotavirus). We did not find any association between the presence of particular pathogens in household animals, and MSD in children.

**Conclusions and significance:**

Public health agencies should continue to promote frequent hand washing, including after animal contact, to reduce the risk of MSD. Future studies should address specific causal relations of MSD with sheep and chicken husbandry practices, and with the presence of rodents.

## Author summary

Diarrheal disease is one of the leading causes of death worldwide in children younger than 5 years. Exposure to animals in homes may be a risk factor for diarrhea in children. To test this, we studied a subset of children in the Global Enteric Multicenter Study (GEMS) in rural western Kenya, whose caretakers reported the presence of animals in the children’s homesteads. In GEMS, children with moderate-to-severe diarrhea (MSD) were matched with children without MSD, who were of the same sex, similar age and who lived in the same area. We asked questions about the presence and management of animals in the children’s homesteads. We also collected fecal specimens from domestic animals present at homesteads and tested these for microbes that could cause diarrheal disease in children. We found that children who reportedly washed their hands after animal contact, and who lived in a homestead with adult sheep that were not confined to a pen overnight, had a lower risk of MSD. Children who lived in homesteads that owned more adult sheep, or in which fresh rodent droppings were observed frequently, had a higher risk of MSD, as did children who reportedly participated in providing water to chickens in the homestead. We did not find any association between the presence of particular pathogens in household animals, and MSD in children.

## Introduction

Diarrheal disease remains among the leading causes of global mortality in children younger than 5 years [1, 2]. Although the mortality rate due to diarrheal disease in this age group in Africa has decreased by nearly 4% per year since 2000, it remains unacceptably high: it is estimated that 12% of deaths in children younger than five years in Africa are due to diarrhea, amounting to almost half a million childhood deaths annually [2]. While mortality rates have decreased, the incidence of diarrheal disease in young children in low- and middle-income countries has shown little change, from 3.4 episodes/child year in 1990 to 2.9 episodes/child year in 2010 [3]. Persistently high incidence rates in these countries are concerning because early childhood diarrhea may have long-term effects on child growth and development [4, 5]. Data characterising risk factors and etiologies of diarrheal disease in children in these settings are important for focusing interventions to decrease associated morbidity and mortality rates.

Many viral, bacterial and protozoal pathogens have been demonstrated as causes of diarrheal disease in children younger than 5 years in developing countries [6]. Contact with domestic animals, including livestock, poultry and companion animals, has been shown to play a role in the epidemiology and transmission to people of a number of these pathogens [7, 8] including *Campylobacter* spp. [9–11], non-typhoidal *Salmonella* [11, 12], diarrheagenic *Escherichia coli* strains [12, 13], *Cryptosporidium* spp. [12–14] and *Giardia duodenalis* [15]. In addition, some reports implicate dogs as a possible source of human infections with unusual strains of rotavirus [16, 17]. Livestock and poultry play a significant role in rural livelihoods in developing countries, providing a variety of benefits to poor households, such as animal-source food (energy-dense food with high biological-value protein, rich in micronutrients), draft power for ploughing and transport, nutrient recycling through manure, income through sale of animals or their products, as well as a form of savings and insurance [18]; however, animal husbandry may also have negative impacts on households, including the transmission of zoonotic and foodborne diseases. In a meta-analysis of demographic health survey data from 30 sub-Saharan African countries examining associations between child health outcomes and household ownership of livestock, Kaur et al [19] found a negative association between livestock and stunting (an indicator of chronic malnutrition), a positive association between livestock and all-cause mortality in children, and no association between livestock and diarrheal illness. In a systematic review and meta-analysis of human diarrhea infections associated with domestic exposure to food-producing animals, Zambrano et al. [20] found consistent evidence of a positive association between exposure and diarrheal illness in people, across a range of animal species and enteric pathogens. Close contact with domestic animals (such as animals sleeping in the house or room) is also associated with impaired growth in children [21, 22]. Considering the potential positive benefits of animal husbandry to rural livelihoods in resource-poor settings, there is a need to identify specific husbandry-related practices associated with diarrheal illness. Such evidence can serve as bases for interventions to reduce transmission of enteric pathogens to household members, especially to children, who are particularly vulnerable to mortality, sequelae and developmental consequences of diarrheal disease. Identifying etiologies of diarrheal illness in household members and concurrent infections in domestic animals may provide further utility for these efforts [23–25].

The Global Enteric Multicenter Study (GEMS), a large-scale case-control study designed to identify the etiology and population-based burden of diarrheal disease in children younger than 5 years in developing countries [6, 26], provided an opportunity to study the association between animal-related exposures and diarrheal illness in household children at a rural site in western Kenya. GEMS was a 3-year, prospective, age-stratified, matched case-control study of moderate-to-severe diarrheal illness in children aged 0–59 months, residing in populations under demographic surveillance at four sites in sub-Saharan Africa and three sites in south Asia. The methodology [26–28] and main findings [29] of GEMS have been published. The GEMS Zoonotic Enteric Diseases (GEMS-ZED) sub-study was conducted among a subset of case children and their matched controls enrolled at one of the six GEMS sentinel health centers in rural western Kenya. The objectives of the GEMS-ZED study were to identify animal-related exposures associated with cases of moderate-to-severe diarrhea (MSD) in children, and to identify the major zoonotic enteric pathogens present in the domestic animals residing in the homesteads of case and control children.

## Materials and methods

### Study site

The GEMS sentinel health center for this study was St Elizabeth Mission Hospital in Lwak (henceforth referred to as Lwak Hospital), located in Rarieda sub-county, Siaya County (formerly Nyanza Province) in western Kenya. Lwak Hospital is the designated referral facility for population-based infectious disease surveillance (PBIDS) conducted in the surrounding 33 villages by the Kenya Medical Research Institute (KEMRI) and the U.S. Centers for Disease Control and Prevention (CDC) [30]. The area also falls within the KEMRI/CDC health and demographic surveillance system (HDSS) site in western Kenya [31]. The HDSS provides general demographic and health information including population age-structure, migration, fertility rates, birth and death rates, verbal autopsy, access and utilization of health care for approximately 220,000 inhabitants in 55,000 households. The primary economic livelihood is subsistence farming and fishing, and an estimated 70% of the population lived below the poverty line in 2003 [32]. The area is culturally homogeneous, with 95% of people being ethnically Luo [33]. Households in the PBIDS villages are clustered into compounds composed of related family units, with most compounds having between one and five family units [33]. Animal husbandry is common: 89% of compounds own at least one species of livestock or poultry, with 86% owning poultry (median flock size: 10), 49% cattle (median herd size: 4), 48% goats (median herd size: 4) and 18% sheep (median herd size: 3) (KEMRI/CDC HDSS data for 2008). Among compounds that own livestock, approximately one-half also own cats and/or dogs (International Emerging Infections Program–Zoonoses Project data for 2009). Rodents, including black rats (*Rattus rattus*), are also commonly present in and around houses in the PBIDS site [34].

### GEMS

From January 31, 2008 through January 29, 2011, children 0–59 months old who sought care at selected sentinel health centers (including Lwak Hospital) and belonged to the HDSS population were screened for diarrhea. To be eligible for inclusion in GEMS, the diarrhea episode had to meet the case definition for MSD [29], which was three or more loose stools within the previous 24 h, with onset within the previous 7 days after a period of at least 7 diarrhea-free days, with one or more of the following: sunken eyes; loss of skin turgor; intravenous rehydration administered or prescribed; dysentery; or hospitalized with diarrhea or dysentery. Each GEMS site restricted enrollment to the first nine eligible cases per age stratum per fortnight. Three age strata were targeted: infants (0–11 months), toddlers (12–23 months), and children (24–59 months). For every enrolled case, one to three children without diarrhea were enrolled as controls. Controls were matched to individual cases by age (within 2 months of age for patients aged 0–23 months, and within 4 months of age for patients aged 24–59 months), sex, and residence (same or nearby village as patient). Potential controls were randomly selected from the KEMRI/CDC HDSS database and enrolled during a home visit within 14 days of the matched case. Potential controls who had diarrhea in the previous 7 days were ineligible. At enrollment, primary caregivers (parent or other caretaker) of cases and controls were interviewed to obtain demographic, epidemiological and clinical information. In addition, each case and control provided at least 3 g of fresh stool, which was submitted to the laboratory for identification of enteric pathogens using standard methods as described by Panchalingam et al. [28].

### GEMS-ZED substudy

The GEMS-ZED substudy collected and analysed additional data on animal-related factors from a subset of GEMS case and matched control children with reported animal presence at home. From November 4, 2009 through February 4, 2011, all cases enrolled into GEMS at Lwak Hospital were screened for inclusion in the GEMS-ZED study. (Enrollment into GEMS continued for a short period after the official end date of January 29, 2011, during which time 3 case-control pairs were enrolled into GEMS-ZED. Data from the GEMS study [laboratory test results and wealth index] are not available for these 3 pairs.) Between zero and six cases per fortnight (median of two) were enrolled into GEMS at Lwak Hospital during the GEMS-ZED study period. Only cases and controls whose primary caregiver reported presence of animals (domestic animals as well as peridomestic wild rodents) at the child’s compound during the GEMS enrollment interview were considered eligible. For each eligible case, the first eligible GEMS-enrolled matched control was identified, resulting in one-to-one matching in the GEMS-ZED dataset. If no eligible child could be identified among the GEMS set of one to three matched controls, then the case was not enrolled into GEMS-ZED. Caregivers of eligible cases and controls were approached for enrollment into the GEMS-ZED study during a separate home visit that took place within 2 weeks of their enrollment into the GEMS study. Written informed consent for participation in the study was sought from the primary caregiver, as well as from the head of the compound of residence of each eligible child; only compounds in which both individuals provided consent were enrolled. Compounds were excluded if the child participating in GEMS had died subsequent to enrollment, or if no domestic animals were found to be resident (for example, if animals had died or were sold subsequent to GEMS enrollment).

Following enrollment, both the head of the compound and the child’s caregiver were interviewed using a standard questionnaire. The questionnaire consisted of two parts: the first part dealt with residence and husbandry of domestic animals in the compound (livestock, poultry, dogs and cats), as well as observations relating to the presence of rodents in and around the compound, and was asked of the person in the compound responsible for the management of animals (typically the head of the compound). The second part dealt with information specific to the participating child, relating to exposures to animals and their environment, and was asked of the child’s caregiver. A summary of the items included in the questionnaire is presented in S1 Table.

At the enrollment visit, fecal specimens were collected from a convenience sample of domestic animals resident at the compound. Specimens from a single species and age category (young, unweaned animals vs. older animals) were pooled together, with specimens from a maximum of five animals collected in a single pool, and a maximum of two pools per species and age category combination (i.e. a maximum of ten animals per species and age category combination were sampled from a compound). A previous study showed good agreement of bacterial culture results between individual and pooled fecal samples of five individuals per pool [35]. Between 3 and 10 g of feces were collected directly from the rectum of larger animals (cattle, sheep, goats and adult dogs). For smaller animals (cats and young dogs), three moistened cotton-tipped swabs were used to collect samples from the animal’s rectum and placed directly into transport media (two in modified Cary Blair and one in buffered glycerol saline); whole feces were not routinely collected from smaller animals.

For poultry, groups of birds of a single species (chickens or ducks) were confined overnight on a sheet of thick plastic. Owners were asked to confine approximately five birds per group, and not more than two groups of birds per species. Fecal specimens from a single pool of animals were mixed in a stool cup. Following thorough mixing of the pooled feces, two cotton-tipped swabs were inserted into the feces and then placed in a vial containing modified Cary Blair transport medium. A third swab was placed in a vial containing buffered glycerol saline. All specimen containers were clearly labelled and placed in a sealed bag in a coolbox with icepacks for transport to the laboratory.

Identification of potentially zoonotic enteric pathogens in animal specimens (*Campylobacter jejuni*, *Campylobacter coli*, non-typhoidal *Salmonella*, diarrheagenic *E*. *coli*, *Cryptosporidium*, *Giardia*, and rotavirus) was carried out using an identical protocol to that described for the human stool specimens tested in GEMS [28]. Briefly, bacterial agents were isolated and identified using conventional culture techniques. Three putative *Escherichia coli* colonies of different morphology types were pooled and analysed by multiplex PCR that detect targets for enterotoxigenic (ETEC), enteroaggregative (EAEC), enteropathogenic (EPEC), and enterohaemorrhagic *E*. *coli* (EHEC). The following gene targets defined each *E*. *coli* pathotype: ETEC (either *eltB* for heat-labile toxin [LT], *estA* for heat-stable toxin [ST], or both), ST-ETEC (either *eltB* and *estA*, or *estA* only), typical EPEC (*bfpA* with or without *eae*), atypical EPEC (*eae* without either *bfpA*, *stx1*, or *stx2*), EAEC (*aatA*, *aaiC*, or both), and EHEC (*eae* with *stx1*, *stx2*, or both, and without *bfpA*). Commercial immunoassays were used to detect rotavirus (ProSpecT Rotavirus kit, Oxoid, Basingstoke, UK), *Giardia* and *Cryptosporidium* spp. (TechLab, Inc., Blacksburg, VA, USA). Immunoassays were only performed on whole fecal specimens of adequate volume (≥ 3 g), and were therefore not completed for the majority of cat specimens, because volumes from this species were often inadequate.

To better understand the zoonotic potential, we genotyped *Cryptosporidium* parasites from immunoassay-positive animal fecal specimens. DNA was extracted from 0.5 ml of fecal specimens using a FastDNA SPIN Kit for Soil (MP Biomedicals, Santa Ana, CA). *Cryptosporidium* species present were differentiated by PCR-restriction fragment length polymorphism (RFLP) analysis of the small subunit (SSU) rRNA gene, and confirmed by DNA sequencing of the PCR products [36].

### Data analysis

Data were analysed using R statistical software version 3.1.3 [37]. We used conditional logistic regression (clogit function applying the exact method in R package ‘survival’ [38]) with one-to-one matching to identify animal-related exposures that were significantly associated with MSD.

Exposure variables were screened for inclusion in the multivariable model using univariable conditional logistic regression. As part of the screening process, each exposure variable was evaluated for potential recoding. Husbandry-related variables for which values were conditional upon residence of the species in question were evaluated and recoded if this made biological sense. For example, the question “Do adult sheep enter the cooking area?’” was conditional on residence of adult sheep in the compound. If no adult sheep were resident, the response was recoded as “No–no adult sheep present” rather than a missing value, and compared against “No–adult sheep present but do not enter cooking area” and “Yes–adult sheep present and enter cooking area”. For these variables, the null state (species not resident) was taken as the reference level. Variables related to exposures of children to animals and their environments were kept as binary variables. For example, the question “Does the child play in an area of the compound where adult sheep defecate?” had one of two responses: ‘no’ if no adult sheep were resident in the compound or adult sheep were resident but the child did not play in the area where they defecated, and ‘yes’ if there were adult sheep resident and the child played in the area where they defecated. For categorical variables with four or more categories, we created new binary variables by combining categories based on frequencies. For example, the original four levels for frequency of observation of rodents or their excreta (never, seldom, often or daily) were dichotomised to never/seldom vs. often/daily. Both the original and new variables were tested in the univariable analysis. Continuous variables (e.g. number of chickens owned) were categorised into three categories [category 1: zero values; category 2: values greater than zero and less than or equal to the median value (excluding zeros); category 3: values greater than the median value (excluding zeros)]; both the original continuous variable and the new categorical variable were assessed in the univariable analysis. Variables with a significant number of missing values (>10% of observations) were discarded. Variables with a Wald test p-value greater than 0.2 on univariable analysis were excluded from further analyses. If both the original and recoded variable had a p-value below the threshold of 0.2, the one with the smaller p-value was retained.

After the univariable screening, we assessed collinearity between the selected exposure variables using condition indices (colldiag function in R package ‘perturb’ [39]). A condition index is a number ranging from 1 to infinity that is computed from data on a set of exposure variables–the higher the condition index, the greater the amount of collinearity [40]. The condition indices were investigated by calculating the variance decomposition proportion (VDP) for each condition index over 30, beginning with the largest. Exposure variables with a VDP >0.5 were considered potentially collinear. In cases where it made biological sense to do so, collinear variables were combined to create a new categorical variable. For example, the collinear variables “Chicken manure used in farm” and “Chicken manure used in the compound” were combined to create a variable “Chicken manure used”. When this did not make biological sense, or when the new variable still exhibited collinearity, the collinear variable with the higher univariable p-value was excluded. Remaining variables were taken forward for consideration in the multivariable conditional logistic regression model.

We compared main effects models using Akaike’s information criterion (AIC), whereby models with a smaller AIC are considered more optimal. We used a forward stepwise regression process to select exposure variables to retain in the model. Missing values were handled through multiple imputation (R package ‘mice’ [41]). Building of the main effects model was stopped when the addition of a variable resulted in an increase in the AIC. We assessed interactions between variables in the final main effects model by adding two-way interaction terms to the model and evaluating their effect on the AIC.

For evaluation of the final model, we identified outliers and influential pairs, using the transformation method described in [42] and applying a Bonferroni outlier test. We computed leverage values and delta *β* statistics to identify influential pairs (in R package ‘car’ [43]). To determine if these pairs were having an undue effect on the model, we refit the model with them omitted.

In GEMS, a wealth index quintile for households was generated by principle component analysis of thirteen household assets [26, 44]. The wealth index quintile was forced into the final model as an ordinal variable to evaluate the potential confounding effect of wealth.

### Ethics statement

The GEMS protocol was approved by the KEMRI Scientific and Ethical Review Committee (protocol no. 1155) and the Institutional Review Board at the University of Maryland, School of Medicine, Baltimore, MD, USA. The Centers for Disease Control and Prevention, Atlanta, GA, USA, formally deferred to the IRB at the University of Maryland for review (protocol no. 5038). Written informed consent was obtained from the parent or primary caretaker of each participant before initiation of study activities. The GEMS-ZED study protocol was approved by the KEMRI Scientific and Ethics Review Unit (protocol no. 1572) and the CDC Institutional Review Board (protocol no. 5683). Written informed consent for participation in the study was provided by the parent or primary caretaker of each participant, as well as from the head of the compound of residence of each participant. Protocols for animal involvement were reviewed and approved by the KEMRI and CDC Institutional Animal Care and Use Committees (protocol no. SSC 1572 and 2088OREMULX, respectively). CDC IACUC protocols comply with the Animal Welfare Act (AWA) regulations promulgated by the United States Department of Agriculture (USDA) under Title 9, Code of Federal Regulations, Parts 1–3 as well as the Public Health Service Policy on Humane Care and Use of Laboratory Animals (PHS Policy) administered by the National Institutes of Health (NIH), Office of Laboratory Animal Welfare (OLAW). In Kenya, all vertebrates are protected under Cap 360 (the Prevention of Cruelty to Animals Act) (1963, revised 1983).

## Results

**Fig 1:**
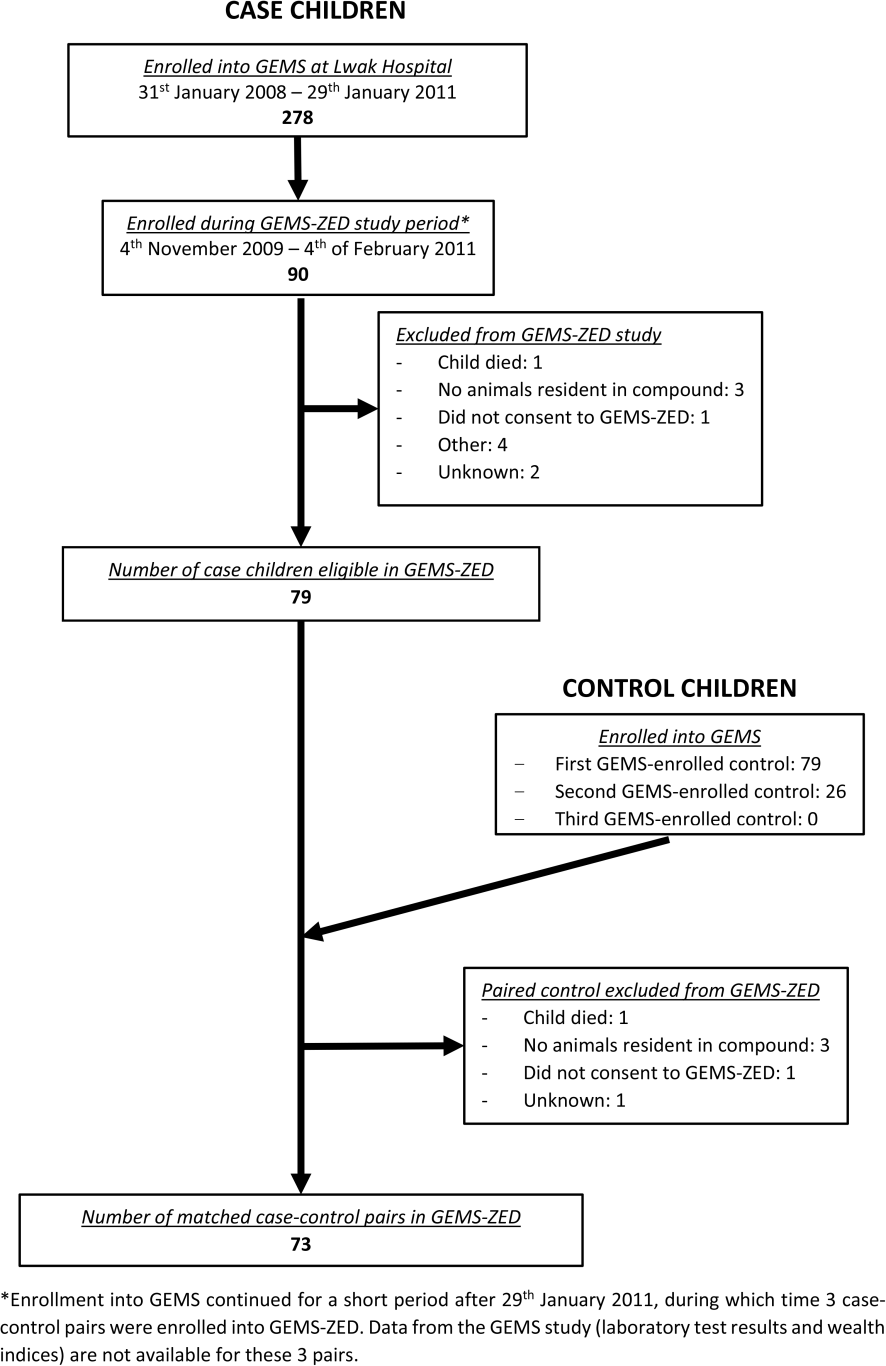
Flow diagram showing selection and enrollment of case and control children into the GEMS-ZED study of moderate-to-severe diarrhea in children in western Kenya.

A flow diagram showing the enrollment of children into the GEMS-ZED study is shown in Fig 1. Of the 90 children with MSD enrolled at Lwak Hospital from November 4th, 2009 through February 4th, 2011, 73 of their households participated in GEMS-ZED, along with 73 control households matched on age, sex and location of the case and control children. The median time between enrollment into GEMS and enrollment into GEMS-ZED was 4 days (range: 0–13 days).

Residence (presence/absence) of particular animal species did not differ significantly between case and control compounds based on the exact McNemar’s test values (Table 1). The wealth index quintile distribution also did not differ between case and control compounds (p = 0.4).

**Table 1:** Ownership of domestic animals by the 73 matched pairs of case-control households enrolled in the GEMS-ZED study of moderate-to-severe diarrhea in children in western Kenya.

Species	Cases	Controls		Exact McNemar’s test p-value
		Owner	Non-owner	
Cattle	Owner	33	16	0.6
	Non-owner	20	4	
Goats	Owner	31	15	1
	Non-owner	16	11	
Sheep	Owner	6	13	0.3
	Non-owner	20	34	
Chickens	Owner	69	2	1
	Non-owner	1	1	
Ducks	Owner	0	4	1
	Non-owner	5	64	
Donkeys	Owner	0	7	0.3
	Non-owner	3	63	
Dogs	Owner	27	12	0.3
	Non-owner	19	15	
Cats	Owner	19	14	0.1
	Non-owner	25	15	

During the screening process, 497 exposure variables were evaluated (including recoded variables). Of these, 100 variables were discarded because they were not applicable or had >10% missing observations. Of the remaining 397 variables, 45 were selected after screening using univariable conditional logistic regression (Wald test p-value ≤ 0.2). Results of the univariable analysis for these variables are presented in S2 Table. After assessment of these variables for collinearity, and combination or exclusion of collinear variables, 37 variables were available for inclusion in the multivariable model (S3 Table). Results of the final model are shown in Table 2. All two-way interactions between variables in the final model were assessed; none resulted in a decrease in the AIC. We also tested for two-way interactions between age group and the main effects in the final model. No interaction terms were significant, meaning that the association between the main effects and MSD did not vary significantly by age group.

**Table 2:** Results of the final multivariable conditional logistic regression model of animal-related factors associated with moderate-to-severe diarrhea in children younger than 5 years in western Kenya (Akaike information criterion: 76.02).

Risk Factor		Matched crude odds ratio (95% CI)	Matched adjusted odds ratio (95% CI)	p-value
**Child washes hands after contact with animals**				
	No	Reference level		
	Yes	0.4 (0.2–0.9)	0.2 (0.08–0.7)	0.008
**Adult sheep sleeping in the pen**				
	No adult sheep	Reference level		
	Adult sheep sleep outside a pen	0.3 (0.1–0.9)	0.1 (0.02–0.5)	0.005
	Adult sheep sleep in pen	2.1 (0.6–6.8)	0.6 (0.09–3.6)	0.6
Total number of sheep*		1.0 (1.0–1.1)	1.2 (1.0–1.5)	0.002
**Fresh rodent excreta (feces/urine) observed outside the house**				
	Never/seldom	Reference level		
	Daily/often	5.0 (1.4–17.3)	7.5 (1.5–37.2)	0.005
**Child's presence during watering the chickens**				
	No	Reference level		
	Yes	2.6 (1.1–5.9)	3.8 (1.2–12.2)	0.02

*Odds ratio for each additional sheep

Variables that were associated with decreased risk of MSD were washing hands after animal contact, and presence of adult sheep that were not confined in a pen overnight. Variables that were associated with increased risk of MSD were increasing number of sheep owned, frequent observation of fresh rodent excreta (feces/urine) outside the house, and participation of the child in providing water to chickens. Inclusion of the wealth index did not result in a substantial change in the log odds ratio of the variables in the final model (<20% change).

In the evaluation of the final model, three pairs were detected as outliers or influential. When we refit the model with these pairs omitted, the same variables as in Table 2 remained in the final model, with the exception that the variable “Adult sheep sleeping in the pen” was replaced by the variable “Distance of sleeping area between child and adult sheep”. Compared with the reference level of no adult sheep, the matched adjusted odds ratio was 0.01 (95% CI 0–0.2) for a distance of 30m or more, and 0.05 (95% CI 0.01–0.04) for a distance of less than 30m.

### Laboratory results

We collected fecal specimens of acceptable quality for diagnostic testing from 2,174 domestic animals of eight species, resulting in a total of 691 pools (median of 5 and range of 1 to 10 pools per compound). Of these, 159 pools (23%) tested positive for one or more potentially zoonotic enteric pathogens (*Campylobacter jejuni*, *C*. *coli*, non-typhoidal *Salmonella*, diarrheagenic *E*. *coli*, *Giardia*, *Cryptosporidium*, or rotavirus). Test results for particular pathogens by host species and age group are given in Table 3. Species with the highest proportion of positive pools for particular pathogens were chickens for *C*. *jejuni* [18/231 (7.8%)] and non-typhoidal *Salmonella* [26/231 (11.3%)]; goats for *C*. *coli* [6/106 (5.7%)]; donkeys for diarrheagenic *E*. *coli* [1/12 (8.3%)]; dogs for *Giardia* [19/69 (27.5%)] and *Cryptosporidium* [4/69 (5.8%)]; and cattle for rotavirus [4/153 (2.6%)].

**Table 3:** Test results for potential zoonotic enteric pathogens in pooled fecal samples collected from domestic animals resident in the homesteads of case and control children enrolled in the GEMS-ZED study of moderate-to-severe diarrhea in children in western Kenya.

*Species*	*Age group*	*Campylobacter jejuni*	*Campylobacter coli*	*Non-typhoidal Salmonella*	*Diarrheagenic Escherichia coli*	*Giardia*	*Cryptosporidium*	*Rotavirus*
*Cattle*	Total	2/155 (2.6%)	1/155 (0.6%)	1/155 (0.6%)	2/149 (1.3%)	8/153 (5.2%)	1/153 (0.6%)	4/153 (2.6%)
	Adults	1/114 (0.9%)	1/114 (0.9%)	1/114 (0.9%)	1/108^a^ (0.9%)	1/113 (0.9%)	0/113 (0%)	1/113 (0.9%)
	Young	1/39 (2.6%)	0/39 (0%)	0/39 (0%)	1/39^b^ (2.6%)	7/38 (18.4%)	1/38 (2.6%)	2/38 (5.3%)
*Goats*	Total	2/106 (1.9%)	6/106 (5.7%)	3/106 (2.8%)	2/81 (2.5%)	5/106 (4.7%)	0/105 (0%)	0/105 (0%)
	Adults	2/91 (2.2%)	4/91 (4.4%)	2/91 (2.2%)	2/72^c^ (2.8%)	5/91 (5.5%)	0/90 (0%)	0/90 (0%)
	Young	0/10 (0%)	1/10 (10%)	0/10 (0%)	0/7 (0%)	0/10 (0%)	0/10 (0%)	0/10 (0%)
*Sheep*	Total	0/54 (0%)	0/54 (0%)	1/54 (1.9%)	2/43 (4.7%)	11/53 (20.8%)	0/53 (0%)	1/53 (1.9%)
	Adults	0/45 (0%)	0/45 (0%)	1/45 (2.2%)	2/36^d^ (5.6%)	6/44 (13.6%)	0/44 (0%)	0/44 (0%)
	Young	0/6 (0%)	0/6 (0%)	0/6 (0%)	0/5 (0%)	4/6 (66.7%)	0/6 (0%)	1/6 (16.7%)
*Chicken*	Total	18/231 (7.8%)	3/231 (1.3%)	26/231 (11.3%)	13/172 (7.6%)	9/224 (4%)	11/224 (4.9%)	3/224 (1.3%)
	Adults	12/141 (8.5%)	3/141 (2.1%)	17/141 (12.1%)	12/108^e^ (11.1%)	8/134 (6%)	4/134 (3%)	3/134 (2.2%)
	Young	2/43 (4.7%)	0/43 (0%)	5/43 (11.6%)	0/31 (0%)	0/43 (0%)	5/43 (11.6%)	0/43 (0%)
*Ducks*	Total	0/5 (0%)	0/5 (0%)	0/5 (0%)	0/5 (0%)	0/5 (0%)	0/5 (0%)	0/5 (0%)
	Adults	0/2 (0%)	0/2 (0%)	0/2 (0%)	0/2 (0%)	0/2 (0%)	0/2 (0%)	0/2 (0%)
	Young	NA	NA	NA	NA	NA	NA	NA
*Donkeys*	Total	0/12 (0%)	0/12 (0%)	0/12 (0%)	1/12 (8.3%)	0/12 (0%)	0/12 (0%)	0/12 (0%)
	Adults	0/10 (0%)	0/10 (0%)	0/10 (0%)	1/10^f^ (10%)	0/10 (0%)	0/10 (0%)	0/10 (0%)
	Young	0/2 (0%)	0/2 (0%)	0/2 (0%)	0/2 (0%)	0/2 (0%)	0/2 (0%)	0/2 (0%)
*Dog*	Total	4/70 (5.7%)	2/70 (4.3%)	5/70 (7.1%)	1/62 (1.6%)	19/69 (27.5%)	4/69 (5.8%)	0/69 (0%)
	Adults	3/69 (4.3%)	2/69 (2.9%)	5/69 (7.2%)	1/62^g^ (1.6%)	19/69 (27.5%)	4/69 (5.8%)	0/69 (0%)
	Young	1/1 (100%)	0/1 (0%)	0/1 (0%)	NA	NA	NA	NA
*Cats*	Total	3/47 (6.4%)	1/47 (2.1%)	4/47 (8.5%)	0/45 (0%)	0/1 (0%)	0/1 (0%)	0/1 (0%)
	Adults	3/43 (7%)	1/47 (2.1%)	4/43 (9.3%)	0/41 (0%)	NA	NA	NA
	Young	0/3 (0%)	0/3 (0%)	0/3 (0%)	0/3 (0%)	NA	NA	NA

Total number of pools may not equal the sum of adult and young pools, due to the presence of some mixed pools of adult and young.

^a^enteroaggregative (EAEC)

^b^enteropathogenic (EPEC)

^c^one EAEC, one EPEC

^d^enterotoxigenic (ETEC)

^e^five ETEC, four EAEC, three EPEC

^f^ETEC

^g^EAEC

Domestic animals from 45/73 (61%) compounds at which a child with MSD resided tested positive to one or more pathogens, compared with 44/73 (60%) compounds with a control child. There were no significant associations on univariable conditional logistic regression between the presence of particular pathogens in domestic animals residing in compounds, and MSD in the participating child from the compound (Table 4). When considering the children’s GEMS laboratory results, we found 21 instances in which the pathogen identified in the child was also identified in one or more species of domestic animals residing in the compound (Table 5).

**Table 4:** Univariable conditional logistic regression results of pathogens identified in domestic animals resident in compounds of children with and without moderate-to-severe diarrhea enrolled in the GEMS-ZED study.

Pathogens identified in resident domestic animals	Status of enrolled children		Matched odds ratio (95% CI)	p-value
	Case (%) n = 73	Control (%) n = 73		
**One or more pathogens identified**	45 (61%)	44 (60%)	1 (0.6–2.0)	0.9
***Giardia***	20 (27%)	20 (27%)	1 (0.5–2.1)	1
**Non-typhoidal *Salmonella***	10 (14%)	19 (26%)	0.4 (0.2–1.1)	0.07
**Diarrheagenic *Escherichia coli***	10 (14%)	10 (14%)	1 (0.4–2.7)	1
***Campylobacter jejuni***	9 (12%)	12 (16%)	0.7 (0.3–1.8)	0.5
***Cryptosporidium***	7 (10%)	7 (10%)	1 (0.4–2.9)	1
***Campylobacter coli***	5 (7%)	7 (10%)	0.6 (0.1–2.5)	0.5
**Rotavirus**	5 (7%)	3 (4%)	1.7 (0.4–7.0)	0.5

**Table 5:** Instances in which a pathogen identified in a child was also identified in one or more species of domestic animals residing in the child’s compound.

Pathogen	Number of positive child-animal pairs	Status of positive child (number of child-animal pairs)	Species of positive animal(s) in household (number of child-animal pairs)
*Giardia*	9	Case (3)	Dog and sheep (1); goat (1), sheep (1)
		Control (6)	Chicken (1); dog (3); goat (1); sheep (1)
Diarrheagenic *Escherichia coli*	4	Case* (3)	Chicken, cattle, goat and sheep (1); chicken, cattle and goat (1); chicken and goat (1)
		Control (1)	Chicken, cattle, dog and goat (1)
Non-typhoidal *Salmonella*	2	Case (1)	Chicken, dog and goat (1)
		Control (1)	Chicken (1)
*Cryptosporidium*	2	Case (2)	Chicken (2)
*Campylobacter jejuni*	2	Case* (2)	Chicken (2)
*Campylobacter coli*	1	Case (1)	Goat (1)
Rotavirus	1	Case (1)	Chicken (1)

*One case child was positive for both diarrheagenic *E*. *coli* and *C*. *jejuni*

Nineteen pooled specimens positive for *Cryptosporidium* spp. by immunoassay were analysed by PCR, including 14 pooled specimens from chickens, 4 from dogs, and 1 from calves. Among them, 7 chicken specimens and the bovine specimen generated the expected PCR products. RFLP analysis indicated the presence of *C*. *meleagridis* in 6 chicken specimens, *C*. *bovis* in one chicken specimen, and *C*. *parvum* in one bovine specimen. None of the canine specimens analysed were positive by PCR.

## Discussion

We identified several animal-related factors associated with MSD in children younger than 5 years from compounds in rural western Kenya in which one or more species of domestic animals were resident. Children who reportedly washed their hands after contact with animals had significantly lower odds of MSD. Water, sanitation, and hygiene (WASH) interventions, including hand washing promotion, are shown to significantly reduce the risks of diarrheal illness in less developed countries [45, 46], but their effectiveness in reducing pathogen exposure specifically from domestic animals in these settings has not been explored. While the protective effect of hand washing has been demonstrated in outbreaks of enteric diseases associated with exposure to domestic animals in public settings [12, 13, 47], in their review Zambrano et al. [20] could find no studies that focused on WASH as a means of limiting disease transmission following domestic exposure to food-producing animals. Our study may be the first to report evidence of a protective effect of hand washing following exposure to household domestic animals in a developing country context. Hand washing after contact with animals may be a reflection of an overall higher frequency of hand washing in these children, and thus the protective effect may extend beyond (or be unrelated to) the risk of diarrheal illness after animal exposure. We recognise that a limitation of our study is reliance on self-reporting of behaviour, including hand washing.

Children from compounds that reported frequent observation of fresh rodent excreta outside the house had significantly higher odds of MSD. In a previous study in the area, a number of rodents were trapped in compounds, including a high proportion of black rats [34]. Rodents, and particularly rats, can be infected with pathogens that cause diarrheal illness in humans [48], including *Salmonella* Typhimurium [49, 50], Shiga-toxin producing *E*. *coli* [51] and *Cryptosporidium parvum* [52, 53]. Fresh rodent feces in areas of the compound may therefore be a source of exposure of children to these pathogens. Absence of rodent excreta could also be a reflection of better sanitation in these compounds, which may be associated with decreased risk of MSD independent of rodents.

Ownership and husbandry of sheep was found to be associated with MSD, but the nature of their role is not clear, with increasing numbers of sheep associated with increased odds, and not confining adult sheep in a pen overnight associated with decreased odds. Distance between children’s sleeping areas and where sheep are kept overnight may also play a role. Sheep are not a common livestock species in the study area, with only 18% of compounds owning sheep (compared with 49% owning cattle and 48% owning goats). Evidence from the literature of a specific role for sheep as risk factors for diarrheal illness in children is scant [54–57]. Consumption of mutton was found to be a risk factor for gastrointestinal illness in children and young adults in Isiolo, eastern Kenya [58]. In our study, we found a low prevalence of potentially zoonotic enteric pathogens in sheep feces (0% - 5%), with the exception of *Giardia* (21%). *Giardia* infection in children was not associated with MSD in GEMS [29].

Participation of the child in providing water to chickens was identified as a risk factor for MSD. In our study, a relatively high proportion of chicken fecal pools were positive for non-typhoidal *Salmonella* (11.3%), *Campylobacter jejuni* (7.8%) and diarrheagenic *E*. *coli* (7.6%). In their meta-analysis of six studies, Zambrano et al. [20] showed that poultry exposure more than doubled the odds of *Campylobacter* spp. infections in humans. Limiting exposure to household poultry, by for example corralling poultry, should therefore reduce the incidence of *Campylobacter* enteritis in children; however, in a randomized study to test this, Oberhelman et al. [59] found that rates of *Campylobacter*-related diarrhea were in fact significantly higher in children from households in which chickens were corralled, compared to those from households in which chickens were not confined. They speculated that this was due to the effect that corralling had on concentrating infected feces in one area, which would increase the risk of exposure to high doses of *Campylobacter* in children who entered corrals. Similarly, in our study we speculate that provision of water to chickens will be carried out mainly in situations where chickens are confined rather than free-ranging, increasing exposure of any accompanying children to enteric pathogens in the accumulated feces; however, we lack more detailed information on the nature of the reported exposure to substantiate this supposition. Active ingestion of chicken feces by infants has been observed in a rural African setting [60], highlighting the risk of zoonotic transmission of enteric pathogens.

In general, the prevalence of potentially zoonotic enteric pathogens in chicken feces in our study was lower than those reported in other studies in comparable settings [9, 24, 59, 61, 62]. Prevalence of zoonotic enteric pathogens in ruminants in our study was also lower when compared with other studies [24, 25, 61–65]. While this may be a reflection of differences in the diagnostic methods used, it could also be due to the extensive, subsistence nature of animal husbandry in our study site and the very small herd/flock sizes. We found no evidence of any association between the presence of particular pathogens in domestic animals and MSD in children, or of infection of children with the same pathogen species, although we note this was a pilot study with a small sample size, which may have limited our ability to detect associations. Enteric pathogens are often shed intermittently in the feces of carrier animals, so it is possible that carrier animals may not have been identified at the time of the specimen collection. The sensitivity of the microbiological methods used in children and in animals is low, as shown by a recent reanalysis of GEMS specimens using quantitative molecular diagnostic methods [66]. Even when the same pathogen species are found in children and in domestic animals in close contact, further characterization often shows genotypic differences between human and animal strains [24, 67, 68], although in some instances further subtyping provides support for zoonotic transmission [69]. In our study, most *Cryptosporidium* species identified from chickens and calves are pathogenic in humans, but further subtyping of species in child and animal specimens is needed to better understand the role of zoonotic transmission in cryptosporidiosis epidemiology.

We tested a large number of animal-related variables for an association with MSD in children. We recognise that with this many variables, significant associations may arise by chance, although the use of AIC in model selection should mitigate this. Furthermore, we do not infer a causal relation from the observed associations. We recommend that our results be used to generate hypotheses of causal links that can be tested in specific studies that address causal relations. These could include the role of sheep, chickens and rodents as risk factors for childhood diarrhea, and the application of WASH interventions to reduce risk. These studies should include established predictors of diarrhea in infants and young children, including breastfeeding and HIV status, in their causal models [70]. Future studies might further examine animal-related factors associated with environmental enteric dysfunction, as a number of zoonotic enteric pathogens have been found to be associated with this condition [71]. The use of quantitative molecular diagnostic methods in well-designed case-control and cohort studies of linked human and animal populations will also be important to understand the role of animals in domestic environments as reservoirs of human enteric pathogens.

## Supporting information

S1 Table Summary of items included in the questionnaire used to interview heads of compounds and caregivers of children enrolled in the GEMS-ZED study. (DOCX)

S2 Table Results of univariable conditional logistic regression analyses. (DOCX)

S3 Table De-identified dataset of the 37 variables included in the multivariable model. (XLSX)
