# Supplementary material for: Animal-related factors associated with moderate-to-severe diarrhea in children younger than five years in western Kenya: A matched case-control study
Source: PLoS Negl Trop Dis. 2017 Aug 4;11(8):e0005795. doi: 10.1371/journal.pntd.0005795 (PMC5559092; doi:10.1371/journal.pntd.0005795)
Supplement: S1 Table — (DOCX) [file pntd.0005795.s001.docx]

**S1 Table. Summary of items included in the questionnaire used to interview heads of compounds and caregivers of children enrolled in the GEMS-ZED study**

| **Residence and husbandry of domestic animals**  Number, age group (adult/young) and sex of resident domestic animals, including cattle, goats, sheep, donkeys, pigs, dogs, cats, chickens, ducks, pigeons, turkeys and others.  Area where animals spend the night; removal and disposal of any manure from this area.  Entry and defecation by animals in the cooking area.  Method of providing drinking water to animals; source of the water; whether source is shared by compound residents.  Production of milk and eggs by animals; consumption of milk and eggs from own animals by compound residents.  Illness in animals within the past 3 weeks.  Frequency and route of administration of any antimicrobials to animals.  Frequency and type of deworming of dogs and cats.  **Presence of peridomestic rodents**  Observation of rodents or fresh rodent excreta in or around the house, outbuildings, crop fields or elsewhere.  **Exposure of child to animals and their environment**  Distance and separation of child’s sleeping area from area where animals spend the night.  Playing in areas where the animals spend the night, or where they defecate; feeding, petting or touching animals; being nuzzled, nibbled or licked by animals.  Helping or accompanying adults in various animal husbandry tasks, including releasing animals from overnight areas, herding, feeding, watering, cleaning, removal of manure, milking, egg collection, treatment, birthing, slaughtering, skinning, butchering.  Washing hands after contact with animals.  Consumption of milk or eggs from compound animals; preparation of milk or eggs before consumption. |
| --- |
