# Supplementary material for: Animal-related factors associated with moderate-to-severe diarrhea in children younger than five years in western Kenya: A matched case-control study
Source: PLoS Negl Trop Dis. 2017 Aug 4;11(8):e0005795. doi: 10.1371/journal.pntd.0005795 (PMC5559092; doi:10.1371/journal.pntd.0005795)
Supplement: S2 Table — (DOCX) [file pntd.0005795.s002.docx]

**S2 Table. Univariable analyses of selected variables**

| Risk Factor | | | Cases | |  | | | Controls | |  | Matched Crude OR (CI) | Wald  p-value |
| --- | --- | --- | --- | --- | --- | --- | --- | --- | --- | --- | --- | --- |
|  |  |  | **Number** | **%** |  | | | **Number** | **%** |  |  |  |
| Young cattle sleeping in the living room | |  |  |  |  | | |  |  |  |  |  |
|  | No young cattle |  | 50 | 68.5 |  | | | 45 | 61.6 |  | Ref | Ref |
|  | Young cattle sleep outside the living room |  | 19 | 26 |  | | | 27 | 37 |  | 0.6 (0.3-1.3) | 0.2 |
|  | Young cattle sleep in the living room |  | 4 | 5.5 |  | | | 1 | 1.4 |  | 3.7 (0.4-33.3) | 0.2 |
|  | Missing Values |  | 0 |  |  | | | 0 |  |  |  |  |
| Young cattle sleeping in the barn | |  |  |  |  | | |  |  |  |  |  |
|  | No young cattle |  | 50 | 68.5 |  | | | 45 | 61.6 |  | Ref | Ref |
|  | Young cattle sleep outside a barn |  | 15 | 20.5 |  | | | 14 | 19.2 |  | 1.1 (0.4-2.5) | 0.9 |
|  | Young cattle sleep in a barn |  | 8 | 11 |  | | | 14 | 19.2 |  | 0.5 (0.2-1.3) | 0.2 |
|  | Missing Values |  | 0 |  |  | | | 0 |  |  |  |  |
| Cattle entered the compound cooking area within the last three weeks | |  |  |  |  | | |  |  |  |  |  |
|  | No |  | 59 | 85.5 |  | | | 55 | 75.3 |  | Ref | Ref |
|  | Yes |  | 10 | 14.5 |  | | | 18 | 24.7 |  | 0.5 (0.2-1.2) | 0.1 |
|  | Missing Values |  | 4 |  |  | | | 0 |  |  |  |  |
| Cattle defecated in the compound cooking area within the last three weeks | |  |  |  |  | | |  |  |  |  |  |
|  | No |  | 63 | 91.3 |  | | | 55 | 76.4 |  | Ref | Ref |
|  | Yes |  | 6 | 8.7 |  | | | 17 | 23.6 |  | 0.2 (0.1-0.8) | 0.02 |
|  | Missing Values |  | 4 |  |  | | | 1 |  |  |  |  |
| Source of water for cattle | |  |  |  |  | | |  |  |  |  |  |
|  | No cattle |  | 24 | 32.9 |  | | | 20 | 27.4 |  | Ref | Ref |
|  | Water is brought to the cattle or the household doesn’t use the same source for drinking |  | 22 | 30.1 |  | | | 33 | 45.2 |  | 0.6 (0.3-1.2) | 0.2 |
|  | The cattle are going to a source of water which is the same used by the household for drinking |  | 27 | 37 |  | | | 20 | 27.4 |  | 1.2 (0.5-2.7) | 0.6 |
|  | Missing Values |  | 0 |  |  | | | 0 |  |  |  |  |
| Owner report at least one clinical sign (diarrhea, lost appetite, weight loss, milk production drop, staring haircoat) in cattle during the last 3 weeks | | | | | | | | | | | |  |
|  | No |  | 48 | 65.8 | |  | 39 | | 53.4 |  | Ref | Ref |
|  | Yes |  | 25 | 34.2 | |  | 34 | | 46.6 |  | 0.6 (0.3-1.2) | 0.2 |
|  | Missing Values |  | 0 |  | |  | 0 | |  |  |  |  |
| Child playing in areas within the compound where adult cattle spend the night | | | | | | | | | | | | |
|  | No |  | 44 | 61.1 | |  | 38 | | 52.1 |  | Ref | Ref |
|  | Yes |  | 28 | 38.9 | |  | 35 | | 47.9 |  | 0.6 (0.3-1.3) | 0.2 |
|  | Missing Values |  | 1 |  | |  | 0 | |  |  |  |  |
| Presence of young goats | |  |  |  | |  |  | |  |  |  |  |
|  | No |  | 52 | 71.2 | |  | 45 | | 61.6 |  | Ref | Ref |
|  | Yes |  | 21 | 28.8 | |  | 28 | | 38.4 |  | 0.6 (0.3-1.3) | 0.2 |
|  | Missing Values |  | 0 |  | |  | 0 | |  |  |  |  |
| Young goats sleeping in the living room | |  |  |  | |  |  | |  |  |  |  |
|  | No young goats |  | 52 | 71.2 | |  | 45 | | 61.6 |  | Ref | Ref |
|  | Young goats sleep outside the living room |  | 20 | 27.4 | |  | 27 | | 37.0 |  | 0.6 (0.2-1.3) | 0.2 |
|  | Young goats sleep in the living room |  | 1 | 1.4 | |  | 1 | | 1.4 |  | 1.0 (0.1-16.0) | 1 |
|  | Missing Values |  | 0 |  | |  | 0 | |  |  |  |  |
| Young goats sleeping in the cooking area | |  |  |  | |  |  | |  |  |  |  |
|  | No young goats |  | 52 | 72.2 | |  | 45 | | 61.6 |  | Ref | Ref |
|  | Young goats sleep outside the cooking area |  | 14 | 19.4 | |  | 24 | | 32.9 |  | 0.4 (0.2-1.1) | 0.07 |
|  | Young goats sleep in the cooking area |  | 6 | 8.3 | |  | 4 | | 5.5 |  | 1 (0.3-3.9) | 1 |
|  | Missing Values |  | 1 |  | |  | 0 | |  |  |  |  |
| Young goats sleeping in the barn | |  |  |  | |  |  | |  |  |  |  |
|  | No young goats |  | 52 | 72.2 | |  | 45 | | 61.6 |  | Ref | Ref |
|  | Young goats do not sleep in a barn |  | 11 | 15.3 | |  | 10 | | 13.7 |  | 0.9 (0.3-2.9) | 0.9 |
|  | Young goats sleep in barn |  | 9 | 12.5 | |  | 18 | | 24.7 |  | 0.3 (0.1-1.0) | 0.05 |
|  | Missing Values |  | 1 |  | |  | 0 | |  |  |  |  |
| Young goats sleeping in the pen | |  |  |  | |  |  | |  |  |  |  |
|  | No young goats |  | 52 | 72.2 | |  | 45 | | 61.6 |  | Ref | Ref |
|  | Young goats do not sleep in a pen |  | 14 | 19.4 | |  | 23 | | 31.5 |  | 0.5 (0.2-1.1) | 0.09 |
|  | Young goats sleep in pen |  | 6 | 8.3 | |  | 5 | | 6.8 |  | 1.0 (0.2-4.6) | 1 |
|  | Missing Values |  | 1 |  | |  | 0 | |  |  |  |  |
| Adult goat sleeping in the barn | |  |  |  | |  |  | |  |  |  |  |
|  | No adult goats |  | 27 | 37 | |  | 26 | | 36.1 |  | Ref | Ref |
|  | Adult goats do not sleep in a barn |  | 24 | 32.9 | |  | 15 | | 20.8 |  | 1.9 (0.7-4.9) | 0.2 |
|  | Adult goats sleep in barn |  | 22 | 30.1 | |  | 31 | | 43.1 |  | 0.6 (0.3-1.5) | 0.3 |
|  | Missing Values |  | 0 |  | |  | 1 | |  |  |  |  |
| Frequency of manure collection from goats | |  |  |  | |  |  | |  |  |  |  |
|  | No manure from young goats |  | 52 | 71.2 | |  | 45 | | 61.6 |  | Ref | Ref |
|  | Collect manure from young goats once or more a day |  | 10 | 13.7 | |  | 17 | | 23.3 |  | 0.5 (0.2-1.2) | 0.1 |
|  | Collect manure from young goats less than once a day |  | 11 | 15.1 | |  | 11 | | 15.1 |  | 0.8 (0.3-2.4) | 0.7 |
|  | Missing Values |  | 0 |  | |  | 0 | |  |  |  |  |
| Owner report at least one clinical sign (diarrhea, lost appetite, weight loss, milk production drop, staring haircoat) in goats during the last 3 weeks | | | | | | | | | | | | |
|  | No |  | 63 | 86.3 | |  | 52 | | 71.2 |  | Ref | Ref |
|  | Yes |  | 10 | 13.7 | |  | 21 | | 28.8 |  | 0.4 (0.2-0.9) | 0.03 |
|  | Missing Values |  | 0 |  | |  | 0 | |  |  |  |  |
| Young sheep sleeping in the barn | |  |  |  | |  |  | |  |  |  |  |
|  | No young sheep |  | 58 | 79.5 | |  | 57 | | 78.1 |  | Ref | Ref |
|  | Young sheep do not sleep in a barn |  | 10 | 13.7 | |  | 4 | | 5.5 |  | 2.6 (0.7-9.7) | 0.2 |
|  | Young sheep sleep in barn |  | 5 | 6.8 | |  | 12 | | 16.4 |  | 0.5 (0.2-1.4) | 0.2 |
|  | Missing Values |  | 0 |  | |  | 0 | |  |  |  |  |
| Young sheep sleeping in the pen | |  |  |  | |  |  | |  |  |  |  |
|  | No young sheep |  | 58 | 79.5 | |  | 57 | | 78.1 |  | Ref | Ref |
|  | Young sheep do not sleep in a pen |  | 5 | 6.8 | |  | 13 | | 17.8 |  | 0.4 (0.2-1.3) | 0.1 |
|  | Young sheep sleep in pen |  | 10 | 13.7 | |  | 3 | | 4.1 |  | 3.8 (0.8-17.8) | 0.09 |
|  | Missing Values |  | 0 |  | |  | 0 | |  |  |  |  |
| Adult sheep sleeping in the barn | |  |  |  | |  |  | |  |  |  |  |
|  | No adult sheep |  | 54 | 75 | |  | 47 | | 64.4 |  | Ref | Ref |
|  | Adult sheep do not sleep in a barn |  | 10 | 13.9 | |  | 6 | | 8.2 |  | 1.7 (0.6-5.0) | 0.4 |
|  | Adult sheep sleep in barn |  | 8 | 11.1 | |  | 20 | | 27.4 |  | 0.3 (0.1-0.9) | 0.03 |
|  | Missing Values |  | 1 |  | |  | 0 | |  |  |  |  |
| Adult sheep sleeping in the pen | |  |  |  | |  |  | |  |  |  |  |
|  | No adult sheep |  | 54 | 74 | |  | 47 | | 64.4 |  | Ref | Ref |
|  | Adult sheep do not sleep in a pen |  | 8 | 11 | |  | 21 | | 28.8 |  | 0.3 (0.1-0.9) | 0.02 |
|  | Adult sheep sleep in pen |  | 11 | 15.1 | |  | 5 | | 6.8 |  | 2.1 (0.6-6.8) | 0.2 |
|  | Missing Values |  | 0 |  | |  | 0 | |  |  |  |  |
| Sheep manure is stored | |  |  |  | |  |  | |  |  |  |  |
|  | No |  | 64 | 87.7 | |  | 58 | | 79.5 |  | Ref | Ref |
|  | Yes |  | 9 | 12.3 | |  | 15 | | 20.5 |  | 0.5 (0.2-1.3) | 0.2 |
|  | Missing Values |  | 0 |  | |  | 0 | |  |  |  |  |
| Total number of sheep | |  |  |  | |  |  | |  |  |  |  |
|  | Total number of sheep |  | Mean=3 | IQR=0.2 | |  | Mean=2 | | IQR=2 |  | 1.0 (1.0-1.1) | 0.2 |
|  | Missing Values |  | 1 |  | |  | 0 | |  |  |  |  |
| Source of water for sheep | |  |  |  | |  |  | |  |  |  |  |
|  | No sheep |  | 54 | 74 | |  | 47 | | 64.4 |  | Ref | Ref |
|  | Water is brought to the sheep or the household doesn’t use the same source for drinking |  | 11 | 15.1 | |  | 21 | | 28.8 |  | 0.5 (0.2-1.1) | 0.07 |
|  | The sheep are going to a source of water which is the same used by the household for drinking |  | 8 | 11 | |  | 5 | | 6.8 |  | 1.4 (0.4-4.2) | 0.6 |
|  | Missing Values |  | 0 |  | |  | 0 | |  |  |  |  |
| Child playing in area within the compound where adult sheep sleep | |  |  |  | |  |  | |  |  |  |  |
|  | No |  | 62 | 84.9 | |  | 54 | | 75 |  | Ref | Ref |
|  | Yes |  | 11 | 15.1 | |  | 18 | | 25 |  | 0.6 (0.2-1.3) | 0.2 |
|  | Missing Values |  | 0 |  | |  | 1 | |  |  |  |  |
| Child playing in area within the compound where adult sheep defecate | |  |  |  | |  |  | |  |  |  |  |
|  | No |  | 61 | 85.9 | |  | 53 | | 72.6 |  | Ref | Ref |
|  | Yes |  | 10 | 14.1 | |  | 20 | | 27.4 |  | 0.4 (0.2-1.0) | 0.06 |
|  | Missing Values |  | 2 |  | |  | 0 | |  |  |  |  |
| Distance of sleeping area between child and sheep | |  |  |  | |  |  | |  |  |  |  |
|  | No adult sheep |  | 54 | 74 | |  | 47 | | 64.4 |  | Ref | Ref |
|  | Distance from where the child sleeps to where the adult sheep sleep is 30 meters or more |  | 11 | 15.1 | |  | 12 | | 16.4 |  | 0.8 (0.3-2.1) | 0.7 |
|  | Distance from where the child sleeps to where the adult sheep sleep is less than 30 meters |  | 8 | 11 | |  | 14 | | 19.2 |  | 0.5 (0.2-1.3) | 0.2 |
|  | Missing Values |  | 0 |  | |  |  | |  |  |  |  |
| The manure of chicken is used in the farm | |  |  |  | |  |  | |  |  |  |  |
|  | No |  | 19 | 26.8 | |  | 8 | | 11.6 |  | Ref | Ref |
|  | Yes |  | 52 | 73.2 | |  | 61 | | 88.4 |  | 0.3 (0.1-0.8) | 0.02 |
|  | Missing Values |  | 2 |  | |  | 4 | |  |  |  |  |
| The manure of chicken is disposed within the compound | |  |  |  | |  |  | |  |  |  |  |
|  | No |  | 57 | 80.3 | |  | 62 | | 89.9 |  | Ref | Ref |
|  | Yes |  | 14 | 19.7 | |  | 7 | | 10.1 |  | 3.3 (0.9-12.1) | 0.07 |
|  | Missing Values |  | 2 |  | |  | 4 | |  |  |  |  |
| Source of water for the chickens is a dam | |  |  |  | |  |  | |  |  |  |  |
|  | No |  | 34 | 47.9 | |  | 41 | | 58.9 |  | Ref | Ref |
|  | Yes |  | 37 | 52.1 | |  | 29 | | 41.4 |  | 1.9 (0.7-4.6) | 0.2 |
|  | Missing Values |  | 2 |  | |  | 3 | |  |  |  |  |
| Child's presence during feeding the chickens | |  |  |  | |  |  | |  |  |  |  |
|  | No |  | 17 | 23.6 | |  | 25 | | 34.7 |  | Ref | Ref |
|  | Yes |  | 55 | 76.4 | |  | 47 | | 65.3 |  | 2.1 (0.9-5.3) | 0.1 |
|  | Missing Values |  | 1 |  | |  | 1 | |  |  |  |  |
| Child's presence during watering the chickens | |  |  |  | |  |  | |  |  |  |  |
|  | No |  | 31 | 43.1 | |  | 43 | | 59.7 |  | Ref | Ref |
|  | Yes |  | 41 | 56.9 | |  | 29 | | 40.3 |  | 2.5 (1.1-5.7) | 0.03 |
|  | Missing Values |  | 1 |  | |  | 1 | |  |  |  |  |
| Child's presence during chicken plucking | |  |  |  | |  |  | |  |  |  |  |
|  | No |  | 38 | 52.8 | |  | 46 | | 63.9 |  | Ref | Ref |
|  | Yes |  | 34 | 47.2 | |  | 26 | | 36.1 |  | 1.7 (0.8-3.4) | 0.2 |
|  | Missing Values |  | 1 |  | |  | 1 | |  |  |  |  |
| Child's presence during chicken butchering | |  |  |  | |  |  | |  |  |  |  |
|  | No |  | 43 | 59.7 | |  | 50 | | 69.4 |  | Ref | Ref |
|  | Yes |  | 29 | 40.3 | |  | 22 | | 30.6 |  | 1.6 (0.8-3.5) | 0.2 |
|  | Missing Values |  | 1 |  | |  | 1 | |  |  |  |  |
| Cat ownership | |  |  |  | |  |  | |  |  |  |  |
|  | No |  | 40 | 54.8 | |  | 29 | | 39.7 |  | Ref | Ref |
|  | Yes |  | 33 | 45.2 | |  | 44 | | 60.3 |  | 0.6 (0.3-1.1) | 0.08 |
|  | Missing Values |  | 0 |  | |  | 0 | |  |  |  |  |
| Presence of adult cat | |  |  |  | |  |  | |  |  |  |  |
|  | No |  | 40 | 54.8 | |  | 29 | | 39.7 |  | Ref | Ref |
|  | Yes |  | 33 | 45.2 | |  | 44 | | 60.3 |  | 0.6 (0.3-1.1) | 0.08 |
|  | Missing Values |  | 0 |  | |  | 0 | |  |  |  |  |
| Adult cat spending the night in the house/living area | |  |  |  | |  |  | |  |  |  |  |
|  | No adult cats |  | 40 | 55.6 | |  | 29 | | 39.7 |  | Ref | Ref |
|  | Present adult cats not spending the night within the house/living area |  | 21 | 29.2 | |  | 32 | | 43.8 |  | 0.5 (0.3-1.0) | 0.07 |
|  | Present adult cats spending the night within the house/living area |  | 11 | 15.3 | |  | 12 | | 16.4 |  | 0.7 (0.2-2.0) | 0.5 |
|  | Missing Values |  | 1 |  | |  | 0 | |  |  |  |  |
| Adult cat free during the night | |  |  |  | |  |  | |  |  |  |  |
|  | No adult cats |  | 40 | 55.6 | |  | 29 | | 40.3 |  | Ref | Ref |
|  | Present adult cats are not free in the compound at night |  | 11 | 15.3 | |  | 12 | | 16.7 |  | 0.6 (0.2-1.9) | 0.4 |
|  | Present adult cats free in the compound at night |  | 21 | 29.2 | |  | 31 | | 43.1 |  | 0.5 (0.3-1.1) | 0.08 |
|  | Missing Values |  | 1 |  | |  | 1 | |  |  |  |  |
| Cats entered the compound cooking area within the last three weeks | |  |  |  | |  |  | |  |  |  |  |
|  | No |  | 42 | 57.5 | |  | 31 | | 42.5 |  | Ref | Ref |
|  | Yes |  | 31 | 42.5 | |  | 42 | | 57.5 |  | 0.6 (0.3-1.1) | 0.08 |
|  | Missing Values |  | 0 |  | |  | 0 | |  |  |  |  |
| Frequency of deworming of cats | |  |  |  | |  |  | |  |  |  |  |
|  | No cats |  | 40 | 54.8 | |  | 29 | | 39.7 |  | Ref | Ref |
|  | The cats are never dewormed |  | 33 | 45.2 | |  | 43 | | 58.9 |  | 0.6 (0.3-1.1) | 0.1 |
|  | The cats are sometimes dewormed |  | 0 | 0 | |  | 1 | | 1.4 |  | NA | 1 |
|  | Missing Values |  | 0 |  | |  | 0 | |  |  |  |  |
| Adult cat nuzzling, nibbling or licking the child | |  |  |  | |  |  | |  |  |  |  |
|  | No |  | 68 | 93.2 | |  | 61 | | 85.9 |  | Ref | Ref |
|  | Yes |  | 5 | 6.8 | |  | 10 | | 14.1 |  | 0.5 (0.2-1.5) | 0.2 |
|  | Missing Values |  | 0 |  | |  | 2 | |  |  |  |  |
| Separation of sleeping areas of the child and adult cats | |  |  |  | |  |  | |  |  |  |  |
|  | No adult cat |  | 40 | 58 | |  | 29 | | 41.4 |  | Ref | Ref |
|  | Sleeping areas of the child and adult cats not separated |  | 4 | 5.8 | |  | 3 | | 4.3 |  | 1.2 (0.2-7.2) | 0.8 |
|  | Sleeping areas of the child and adult cats separated |  | 25 | 36.2 | |  | 38 | | 54.3 |  | 0.5 (0.3-1.1) | 0.08 |
|  | Missing Values |  | 4 |  | |  | 3 | |  |  |  |  |
| Distance between sleeping areas of the child and adult cats | |  |  |  | |  |  | |  |  |  |  |
|  | No adult cat |  | 40 | 58 | |  | 29 | | 41.4 |  | Ref | Ref |
|  | Distance from where the child sleeps to where the adult cats sleep is 4 meters or more |  | 14 | 20.3 | |  | 22 | | 31.4 |  | 0.5 (0.2-1.1) | 0.1 |
|  | Distance from where the child sleeps to where the adult cats sleep is less than 4 meters |  | 15 | 21.7 | |  | 19 | | 27.1 |  | 0.6 (0.3-1.4) | 0.2 |
|  | Missing Values |  | 4 |  | |  | 3 | |  |  |  |  |
| Child washes hands after contact with animals | |  |  |  | |  |  | |  |  |  |  |
|  | No |  | 32 | 48.5 | |  | 22 | | 32.4 |  | Ref | Ref |
|  | Yes |  | 34 | 51.5 | |  | 46 | | 67.6 |  | 0.5 (0.2-1.2) | 0.1 |
|  | Missing Values |  | 7 |  | |  | 5 | |  |  |  |  |
| Frequency of fresh rodents excreta in the house | |  |  |  | |  |  | |  |  |  |  |
|  | Never |  | 41 | 56.2 | |  | 2 | | 2.7 |  | Ref | Ref |
|  | Seldom |  | 18 | 24.7 | |  | 9 | | 12.3 |  | 2.4 (1.0-5.6) | 0.04 |
|  | Often |  | 8 | 11 | |  | 6 | | 8.2 |  | 1.8 (0.6-5.5) | 0.3 |
|  | Daily |  | 6 | 8.2 | |  | 56 | | 76.7 |  | 4.2 (0.8-21.9) | 0.09 |
|  | Missing Values |  | 0 |  | |  | 0 | |  |  |  |  |
| Fewer than 5 rodents present outside daily or often around the house | |  |  |  | |  |  | |  |  |  |  |
|  | No |  | 58 | 79.5 | |  | 67 | | 91.8 |  | Ref | Ref |
|  | Yes |  | 15 | 20.5 | |  | 6 | | 8.2 |  | 2.8 (1.0-7.8) | 0.05 |
|  | Missing Values |  | 0 |  | |  | 0 | |  |  |  |  |
| Fresh rodent excreta (feces/urine) found daily or often outside the house | |  |  |  | |  |  | |  |  |  |  |
|  | No |  | 58 | 79.5 | |  | 70 | | 95.9 |  | Ref | Ref |
|  | Yes |  | 15 | 20.5 | |  | 3 | | 4.1 |  | 5 (1.4-17.3) | 0.01 |
|  | Missing Values |  | 0 |  | |  | 0 | |  |  |  |  |
